# Observation of photonic Peierls transition for manipulating microwave in metallic diaphragm-array periodic structures

**DOI:** 10.1038/s41598-023-42218-7

**Published:** 2023-09-22

**Authors:** Chia Ho Wu, Chengyang Liu, Xianqing Lin, Wei Wang, Yi Chun Guo, Zhuoyuan Wang, Guoqiang Ye, Fang He, Donghua Ni, Xiaolong Wang, Linfang Shen, Jianqi Shen, Zhengbing Cai, Gang Chen

**Affiliations:** 1 Department of Applied Physics, Zhejiang University of Technology, Hangzhou, 310023 China; 2 Department of Electrical Engineering, Chung Hua University, Hsinchu, 30012 Taiwan; 3 Electronic and Information Engineering College, Ningbo University of Technology, Ningbo, 315000 China; 4 Zhejiang Zhaolong Interconnect Technology Co., Ltd, Deqing, Huzhou, 313200 China; 5 Key Laboratory of Quantum Precision Measurement of Zhejiang Province, College of Science, Zhejiang University of Technology, Hangzhou, 310023 China; 6 Centre for Optical and Electromagnetic Research, College of Optical Science and Engineering, Zhejiang University, East Building No. 5, Zijingang Campus, Hangzhou, 310058 China; 7 Hangzhou Taiding Test Technologies Co., Ltd., Binjiang, Hangzhou, 310051 China

**Keywords:** Materials science, Optics and photonics, Physics

## Abstract

Peierls transition that modifies electronic band structure has attracted intensive attention in solid state physics. In the present work, we report that a photonic analog of Peierls transition has been observed in a 1-D triangular metal diaphragm array, where the photonic bandgap structures have been designed at will by adjusting periodically metal diaphragm positions. It is shown by the numerical analysis that the transmission and radiation effect of the present periodic metal structure designed through the Peierls transition rule exhibits the behavior significantly different from an original periodic structure with each unit cell containing a metal diaphragm. The near- and far-field measurement results are in good agreement with our theoretical simulation. The present effect of photonic Peierls transition can serve as a working mechanism for designing new types of guided wave devices. It can be seen that the photonic Peierls transition would be one of the simplest ways for modifying the transport characteristics of electromagnetic waves in periodic structures.

## Introduction

Since dielectric constants of metal conductors in optical frequency bands have large negative real parts, it can be found by using the Maxwell equations that metal surfaces can support a kind of special electromagnetic modes, namely, surface plasmon polaritons^1^. A mode of surface plasmon polaritons is an electromagnetic wave propagating at the interface between a conductor and a dielectric medium. This electromagnetic mode is a coupled state between electromagnetic waves and free electrons in a conductor, and its polarization is in a TM mode^1^. Such an electromagnetic mode can constrain their electromagnetic energy firmly to the conductor surfaces, and thus arouses the interest of researchers in the field of optical frequency bands. According to the numerical analysis, some new metal guided wave structures in Refs. ^2,3^ have been designed based on the mechanism of surface plasmon polaritons that can highly compress the electromagnetic fields into metal channels with a size much smaller than their wavelength. They also have obvious advantages in improving the density of integrated photonic circuits. However, on the other hand, high confinement of electromagnetic fields to metal surfaces results in high-intensity losses and a drastic reduction in signal transmission distance. In the low frequency bands (e.g., terahertz or microwave band), it is more urgent than in optical bands for circuit systems to confine the electromagnetic fields into transmission lines in a subwavelength manner. Since the metal surface plasma frequency is usually in the ultraviolet band, the concept of surface plasmon polaritons cannot be directly used for effectively constraining the electromagnetic fields and avoiding the electromagnetic crosstalk between circuits in microwave regime. Thus, it seems unrealistic to employ the mechanism of surface plasmon polaritons in the microwave frequency band to develop new efficient transmission lines with confined electromagnetic fields. However, there was a turnaround in 2004, when Pendry et al.^4,5^ proposed a new scenario to effectively trap the electromagnetic fields in the low frequency bands. As is known, the property of metal conductors for confining electromagnetic fields can be enhanced by introducing certain subwavelength periodic ripples on metal surfaces. Moreover, the dispersion characteristics of their electromagnetic modes^4,5^ are very similar to the surface plasmon polaritons of metal-dielectric interfaces in the optical band, and such modes known as spoof surface plasmon polaritons can be used to simulate the transmission characteristics of surface plasmon polaritons in the low frequency band. Such new spoof surface plasmon polaritons are more flexible than traditional ones in practical applications. As the size and shape of unit cells of metallic periodic structures can be adjusted according to the demand at the actual transmission frequencies, spoof surface plasmon polaritons can be realized on metal surfaces in a wide frequency range (from microwave to infrared frequencies)^6^. In order to catch the electromagnetic fields on conductor surfaces efficiently, the textures of periodic structures can be deepened on the conductor surfaces. For example, Hibbins et al.^7^ first realized a 2-D subwavelength periodic copper tube array for constraining the electromagnetic fields in the microwave frequency band. They found that the measured data are in line with the theoretical calculations, according to the dispersion characteristics of 2-D periodic copper tubes measured through experiments. Then this concept was soon introduced into the THz band for designing new waveguide structures. Maier^8^ used periodic metal cylinder structures to design monometallic wire waveguides of electromagnetic fields with strong trap abilities, and calculated dispersion curves and mode field distribution at different periodic lattice constants and groove depths by numerical method, aiming at designing super focused guided-wave structures. Apart from the high confinement, the loss of spoof surface plasmon polaritons should also be considered, because the metal conductor loss related to signal transmission distance is extremely important for the practicability in increasing the propagation length of spoof surface plasmon polaritons. For example, Shen et al. have discussed the loss of 2-D metallic periodic structures in highly constrained electromagnetic fields^9^. In order to improve terahertz waveguide structures, the metal domino-based integrated circuits in the THz band have been proposed^10^. In order that the field can be further confined, a transmission line has been suggested with wedge-shaped metal in unit cells of periodic structures^11^. Then it is easier to control the transmission of electromagnetic waves with such spoof-surface-plasmon-polaritons waveguides than with previous THz waveguides^12^.

Some new waveguides with the same guided wave mechanisms as those in Refs.^8–11^ are suitable for the microwave frequency band, as reported in Refs.^13,14^, where the dispersion characteristics and field distribution of these metallic periodic structures were studied in detail and the experimental measurements were presented. Based on the work of Ref.^15^, the band-pass filter components based on metal dominoes were designed, and the waveguide transmission coefficients were directly measured by the vector network analyzer. Some studies applied the concept of spoof surface plasmon polaritons into traditional planar circuits, and designed transmission circuits with low crosstalk for adjacent circuits by using the high confinement of spoof surface plasmon polaritons transmitted by metallic periodic structures^16–21^. Some novel applications, e.g., design of leaky wave antennas based on spoof surface plasmon plartions, have also been reported in detail^22^. Recently, many concepts based on energy band and topological states in solid state physics have been applied to the design of photonic devices, such as photonic crystals and photonic topological loops^22,23^. The ongoing search in applying the concepts associated with solid state physics to the photonic device design continues to be a hot topic. As far as a linear chain of atoms is concerned, there is an important physical phenomenon in solid state physics called Peierls transition^24^. By periodically moving the atoms slightly, one can narrow the width of a first Brillouin zone and introduce required band gaps into the original linear chain of atoms. The modification to the band structure of electrons in the linear chain of atoms in this way is called the Peierls transition^24^. In a similar manner, the transmission characteristics of electromagnetic waves can also be altered through the so-called photonic Peierls transition by adjusting the periodic metal structures with one-dimensional periodic arrangements.

In the first part of the present work, the dispersion relation of the propagation modes of a 1-D triangular metal diaphragm array on metal surfaces was studied. In order to investigate the characteristics of such periodic structures, which are different from the original ones, adjacent metal diaphragms in the 1-D periodic metal array structure were staggered by a certain distance. Hence, the lattice constants became twice as much as those of the original periodic structure, which can be referred to as “a photonic analog of Peierls transition”. In this case, the range of the first Brillouin zone was halved, and a unit cell was changed from a triangular metal diaphragm into an array structure of two staggered triangular metal diaphragms. This study investigated the basic modes of two triangular periodic metal diaphragm structures, and theoretically explored the dispersion curves, propagation characteristics, and electromagnetic field distribution in the two metallic periodic structures (before and after the photonic Peierls transition). In the second part, the above two metallic periodic structures were fabricated by aluminum for investigating the transmission bandwidth and radiation characteristics of the two metal waveguides. It was found that the staggered triangular metal diaphragm array structures could provide dual-beam scanning in the leaky-wave frequency band. Such changes in lattice constants of periodic metal diaphragm array structures would make the design of new devices more flexible than the original ones. In the present subject of introducing the concept of Peierls transition into one-dimensional metal periodic structures, although the relative displacement of adjacent metal diaphragms in one-dimensional metal periodic structures changes little, the mode transmission characteristics vary much due to change in the numbers of metal diaphragms in a cell of the periodic structure. As a result, for example, a bandgap structure would be introduced into the frequency range of transmission, or a guided mode would be transformed to a leaky wave mode. All such modifications in electromagnetic responses require only a tiny displacement between adjacent metal diaphragms. In general, seen from a perspective of physical intuition, the relative displacement between the two adjacent metal diaphragms is so small that it would be difficult to see that there are significant changes in transmission characteristics. In the present electromagnetic analog of Peierls transition, however, such interesting significant modifications in transmission characteristics occur.

## Results

### Theoretical analysis

Let us consider the topic of transmission characteristics analysis of 1-D triangular periodic metal diaphragm array structures, where we shall transplant the mechanism of Peierls transition in solid state physics to photonics, i.e., the lattice constant changes in integer times. As far as a linear chain of atoms in a solid is concerned, by periodically moving the atoms slightly, one can narrow the width of the first Brillouin zone and introduce the required band gaps into the original linear chain of atoms. Such a modification to the band structure of electrons in the linear chain of atoms in this way is called the Peierls transition^24^. In a similar manner, the transmission characteristics of electromagnetic waves can also be altered through the so-called photonic Peierls transition by adjusting the artificial periodic metal structures with one-dimensional arrangements. In the linear atomic chain with one-dimensional infinite extension, the first Brillouin zone of electrons in the linear atomic chain with the lattice constant $d$ (each cell contains one atom) is in the range $- \pi /d < \beta < \pi /d$. If the position of the $r$-th atom along the chain is shifted slightly (e.g., moving up or down in the perpendicular direction of the chain), the translation symmetry of one-dimensional atomic chain is broken, and each cell containing one atom will now become a new unit cell that contains $r$ atoms. Then the first Brillouin zone will be in the new wavenumber range. Peierls transition is a method to change the structure of one-dimensional atomic energy bands by periodically adjusting the positions of atoms to change the electronic bandgap structure. In this process, as a result, phase transition from conductor to insulator of one-dimensional atomic arrays can occur. By analogy, a photonic bandgap structure in one-dimensional metal periodic structures can also be achieved in the similar manner by changing the cells in a periodic electromagnetic material structure. We expect that the photonic Peierls transition would find two kinds of applications in metal periodic structures: The first one is that it can serve as a working mechanism of filters within a specified frequency range determined by the structure of a unit cell; The second one is that it can lead to the frequency ranges of leakage waves, which is determined by the number of metal diaphragms in a unit cell, and a high directivity scanning antenna can therefore be designed. It is worth mentioning that there are many phase transitions in solid state physics, such as the transition from a non-superconducting state to a superconducting state for superconductor materials, and magnetic material structure change from disorder to order in the process of magnetization. For phase transitions relevant to energy bands, the band structure can be changed by squeezing the space where the lattice is located, but the number of atoms contained in the unit cell does not change. Peierls transition is a method to change the band structure of one-dimensional materials, which involves the change of the number of atoms in a unit cell.

**Figure 1:**
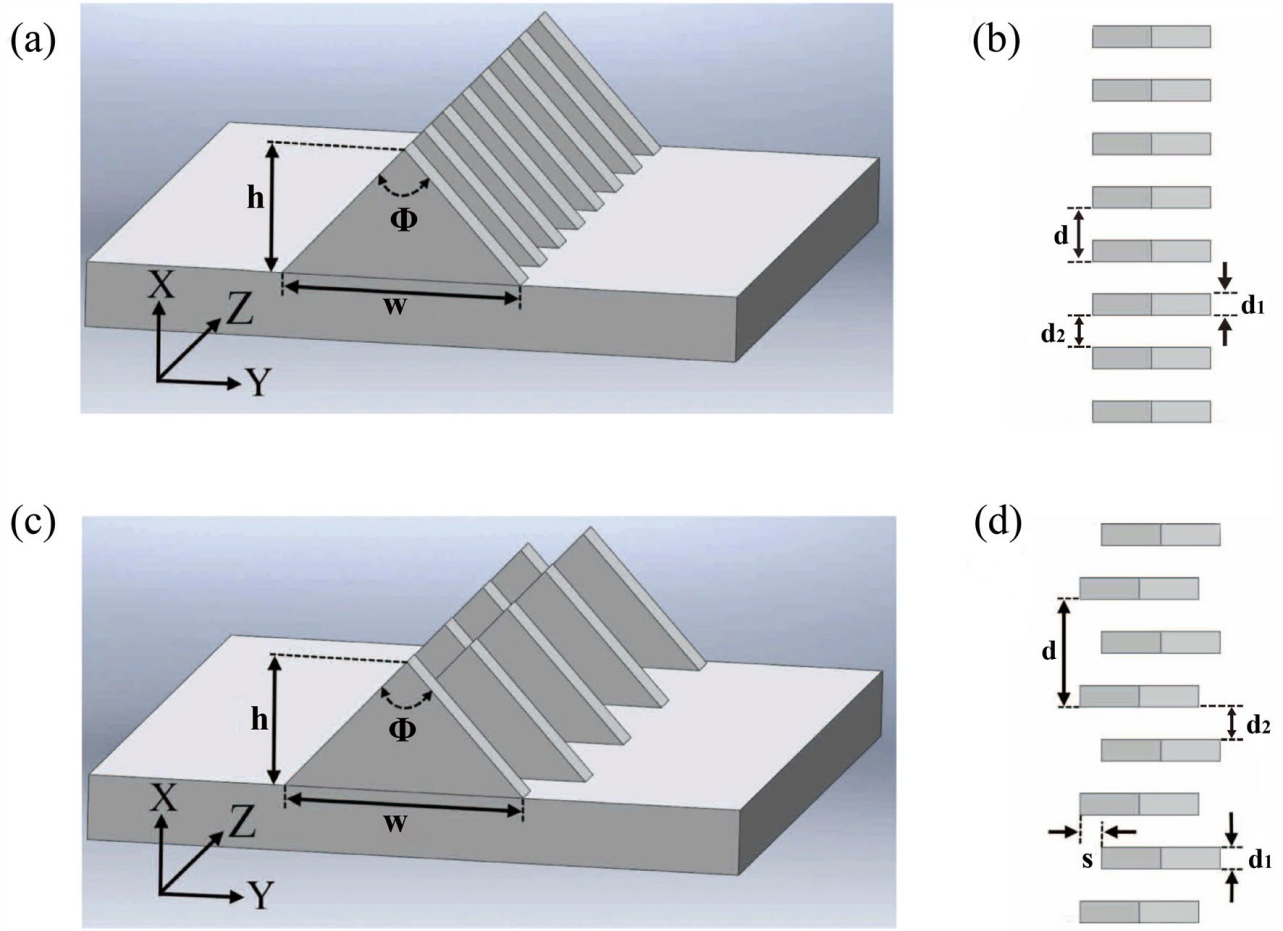
The diagram of unit cells of 1-D triangular periodic metal diaphragm array structures: (**a**) a 1-D triangular periodic metal array structure with the lattice constant $d$; (**b**) the top view of (**a**) for the schematic diagram; (**c**) a 1-D staggered triangular periodic metal structure with the lattice constant $d$; (**d**) the top view of (**c**).

**Figure 2:**
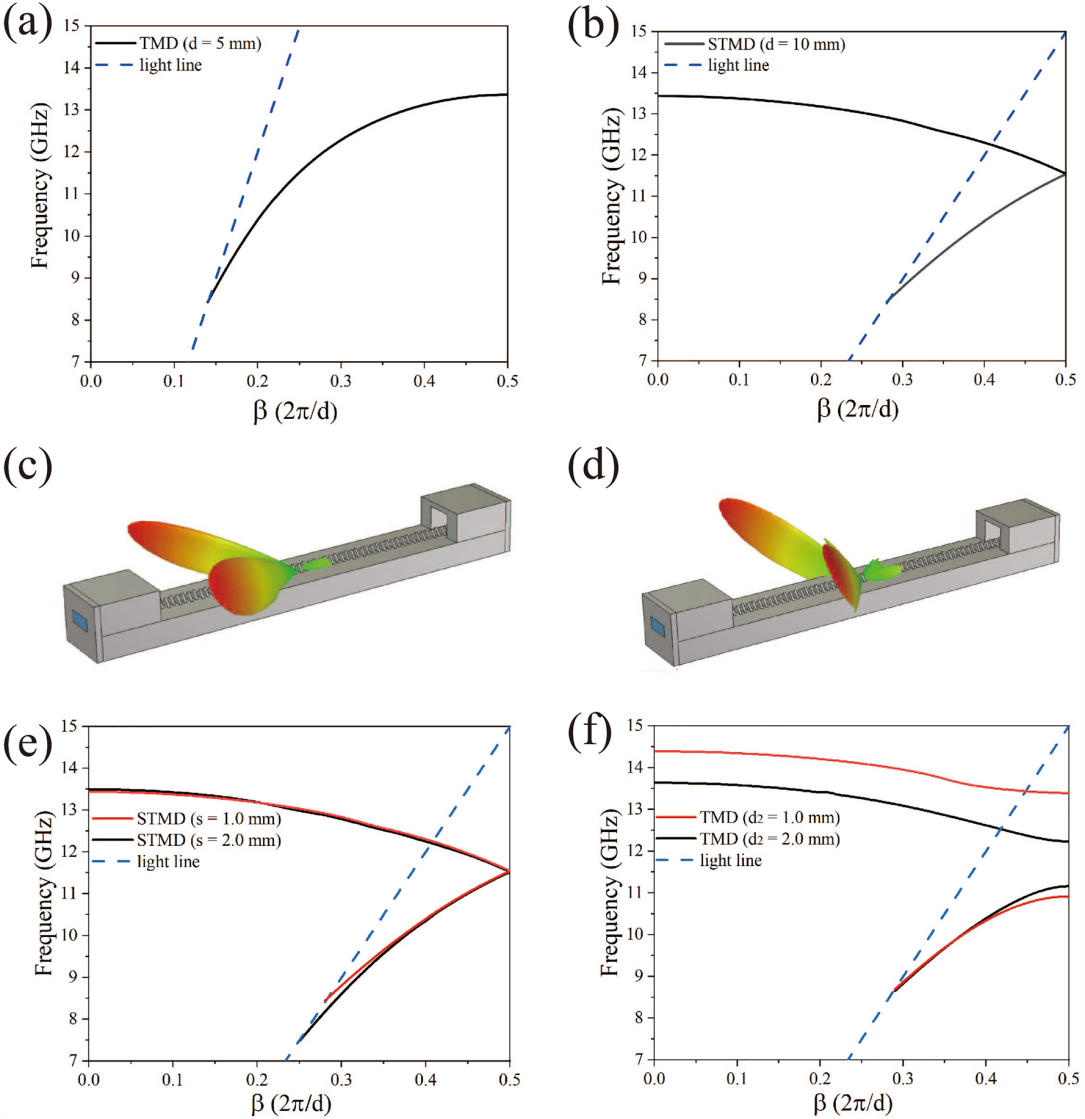
The dispersion curves and far-field distribution of leaky mode: (**a**) the dispersion curves of a 1-D triangular metal diaphragm array with lattice constant $d$ = 5.0 mm; (**b**) the dispersion curves of a 1-D staggered triangular metal diaphragm array with lattice constant $d$ = 10.0 mm; (**c**) the far-field distribution of leaky mode at frequency $f$ = 12.50 GHz; (**d**) the far-field distribution of leaky mode at frequency $f$ = 12.90 GHz; (**e**) the influence of the relative displacement $s$ between the adjacent metal diaphragms on the dispersion curve; (**f**) the influence of the relative displacement between the adjacent metal diaphragms along the longitudinal direction on the dispersion curve.

The two types of plasmonic waveguides considered in this work are presented in Fig. 1. The triangular metal sheets shown in Fig. 1a for our experiment were arranged along a straight line on a metal plate in a waveguide structure. The frequency band, in which the metallic periodic structures enable to allow constraining the electromagnetic fields, can be adjusted by changing the size and shape of the unit cells. In Fig. 1a there is a metallic periodic structure with the lattice constant $d$, of which the geometric parameters of the triangular metal diaphragm include the bottom width $w$, the height $h$, the metal diaphragm thickness $d_{1}$ and the apex angle $\Phi$. The top view of the structure is shown in Fig. 1b. The transmission of waveguides similar to that in Fig. 1a in the THz band has been discussed in Refs.^11,25^, demonstrating that the low-frequency spoof surface plasmon polaritons can be transmitted and there is a strong confinement effect on the electromagnetic fields. The structure of the plasmonic waveguide in Fig. 1c was formed by staggering the bottoms of adjacent triangular metal diaphragms by a short distance $s$ in the plasmonic waveguide given in Fig. 1a. According to the Peierls transition, due to the small displacement distance $s$ mentioned above, the lattice constant of the periodic structure in Fig. 1c was twice as much as that of the structure in Fig. 1a. It must be noted that the transmission characteristics of the two periodic structures have been significantly modified while the displacement distance $s$ was still small. In Fig. 2a, we show the dispersion curves of the 1-D triangular metal diaphragm array (TMDA) plasmonic waveguide structure in Fig. 1a. The dispersion characteristics of the waveguide structure plotted in the first Brillouin zone have been solved by COMSOL (finite element method) under the periodic boundary conditions. Here, the waveguide geometric parameters are given by $d$ = 5.0 mm, $w$ = 11.0 mm, $h$ = 6.0 mm, $d_{1}$ = 2.0 mm and $\Phi$ = 85.02°, which were selected for the waveguide structure given in Fig. 1a.

Here, only one propagation mode in the frequency range (X-band) was considered in Fig. 2a for the entire triangular metal diaphragm periodic structure. In our calculation process, it was assumed that the waveguide was made of perfect conductor materials without ohmic loss. In the model, the cutoff frequency was $f_{TC1}$ = 8.43198 GHz and the asymptotic frequency was $f_{TS1}$ = 13.3650 GHz ($\beta = \pi /d$). The transmission bandwidth of the metallic periodic structure was 4.93302 GHz. To explore new physical phenomena from the above structures, the following efforts have been made in this study: Without varying the size of triangular metal diaphragms, we only change the relative displacement to $s$ = 1.0 mm between two adjacent metal diaphragms in the periodic structure shown in Fig. 1a (here the relative displacement $s$ must be much smaller than the bottom width $w$). In this case, the structure became the staggered triangular metal diaphragm (STMD) periodic one as shown in Fig. 1c, and its top view is illustrated at the right side of the structure in Fig. 1d. A comparison between the top views shows that there were small changes between the two periodic structures in Fig. 1a,c, namely, the lattice constant of Fig. 1c was twice as much as that of Fig. 1a, i.e., $\ d$ = 10.0 mm. The actual wavelength considered ranges from 2.00 to 3.75 cm, and the reference wavelength is selected as 2.60 cm (i.e., the wavelength corresponding to the bandgap edge). In Fig. 2b we present the dispersion curves of the metallic periodic structures for Fig. 1c, which was calculated by the finite element method. These dispersion curves were plotted in the first Brillouin zone as shown in Fig. 2b. Because the lattice constant has changed from $d$ = 5.0 mm in Fig. 1a to $d$ = 10.0 mm in Fig. 1c, the whole metallic periodic structure of Fig. 1c had an extra dispersion curve with a negative slope compared with that of Fig. 1a. For the first dispersion curve, i.e., the black solid line in Fig. 2b, the cutoff frequency was $f_{STC}$ = 8.4380 GHz, the first asymptotic frequency was $f_{STS1}$ = 11.5334 GHz ($\beta = \pi /d$), and the transmission frequency was 3.0954 GHz. The second and third asymptotic frequencies were $f_{STS2}$ = 11.5495 GHz ($\beta = \pi /d$) and $f_{STS3}$ = 13.4372 GHz ($\beta = 0$), respectively. After the second asymptotic frequency, it can be seen that the propagation constant $\beta$ gradually decreases as the frequency increases, and thus, this dispersion curve has a negative slope, and then the negative dispersion curve passes through the light line at $f$ = 12.2554 GHz and enters the radiation zone, becoming a leaky-wave mode until $f$ = 13.4370 GHz $(\beta = 0)$. This indicates that by a small relative displacement between adjacent triangular metal diaphragms, the lattice constant of the triangular periodic metal diaphragm structure could be changed from 5.0 to 10.0 mm and would give rise to the negative slope dispersion. It is worth noting that the second asymptotic frequency in Fig. 2b is almost the same as the third asymptotic frequency, i.e., the band gap between the two modes almost disappears. As a result, the transmission bandwidth of the waveguide increases from 3.0954 to 3.8174 GHz. In this frequency range, this extremely small band gap had almost no effect on the wave propagation. With these data, the negative dispersion curve in Fig. 2b can be easily understood from the dispersion curve in Fig. 2a. The width of the Brillouin zone of Fig. 1a was halved as the lattice constant of Fig. 1c was doubled. The cause of negative dispersion lies in that the dispersion curve in Fig. 2a returns in the direction of decreasing $\beta$ after passing through the point $\beta = 0.25 \times (2\pi /d)$, and the frequency corresponding to $\beta = 0.25 \times (2\pi /d)$ was $f$ = 11.5201 GHz in the dispersion curve of Fig. 2a. When the propagation constant of the dispersion curve goes beyond more than half the width of the first Brillouin zone of the metallic periodic structure in Fig. 2a, it will return to the first Brillouin zone and become a negative dispersion curve. Therefore, the first mode would directly be converted to the second mode after the asymptotic frequency was exceeded. The propagation constant of the staggered triangular periodic metal diaphragm structure in the radiation zone becomes a complex number $k_{z} = \beta + j\alpha_{leaky}$, where $\alpha_{leaky}$ represents the imaginary part of the propagation constant of the leaky wave mode. As a result, the staggered triangular metal diaphragm array structures can provide the frequency-controlled scanning beams in the leaky wave frequency range, and it is necessary to numerically manifest the way in which the elevation and azimuth angles of the main beam change with frequency. The far-field distribution of the staggered triangular periodic metal diaphragm structures in the leaky wave region is shown in Fig. 2c at 12.5 GHz and Fig. 2d at 12.9 GHz. The angle between the two beams is represented by the azimuth difference $\Delta \varphi$, and the elevation angle between each of the two main beams and the horizontal direction is represented by $\theta$. The elevation angle $\theta$ of the beam becomes large gradually with the increasing frequency of the incident wave, and so does the azimuth $\Delta \varphi$ of the beam. For a detailed analysis of the leakage properties of the periodic structures, readers can be referred to Refs.^26,27^. Since the two adjacent triangular metal diaphragms can be regarded as a groove and the electromagnetic waves are radiated from each groove, the expression for the electric field can be written as the contribution of a magnetic current of $N$ grooves to the radiation^28^

1 $$E = E_{m} F(\theta_{c} ,\phi_{c} )\left[ {\frac{{e^{{ - j\beta R_{0} }} }}{{R_{0} }} + \frac{{e^{j\xi } e^{ - \alpha d} e^{{ - j\beta R_{1} }} }}{{R_{1} }} + \cdots } \right]$$

where $E_{m}$ is the amplitude function, $F{(}\theta_{c} ,\phi_{c} {)}$ is the orientation pattern function of the antenna element, $\theta_{c}$ is the elevation angle and $\phi_{c}$ is the azimuth angle of the triangle slope, and the parameter $\xi$ represents the phase angle of the two adjacent antenna elements and $\alpha$ denotes the imaginary part of the propagation constant. For an infinitely long leaky wave antenna, the direction pattern of its radiation field $R{(}\theta_{c} )$ can be expressed as^26^

2 $$R(\theta_{c} ) \approx \frac{{\cos^{2} \theta_{c} }}{{\left( {\alpha /k_{0} } \right)^{2} + \left( {\beta /k_{0} - \sin \theta } \right)^{2} }}$$

Considering that the plasmonic waveguide can provide a beam with frequency scanning, the electromagnetic wave can be incident in the direction of the beam scanning, which will implement a concept similar to the multi-layer Brewster angle. In Fig. 2e, we show the variation of the dispersion curve when the metal diaphragm moves laterally in the metal periodic structure of Fig. 1c and it can be seen that the relative position of the two adjacent metal diaphragms changes the first and second asymptotic frequencies. In Fig. 2f, it demonstrates the result of the variation of the dispersion curve of two adjacent metal diaphragms moving forward or backward in the chain direction of the periodic metal structure plotted in Fig. 1c, where the lattice constant remains invariant. It should be pointed out that when the positions of the metal diaphragms move forward or backward (in the chain direction) by less than 10% of the metal diaphragm width, it will lead to an additional bandgap structure for the electromagnetic waves. Such a behavior can be used in design of a low-pass filter. From the analysis results of the references^15, 29^, it is found that the dispersion curve of the present metal periodic structure extending from the cutoff frequency to the asymptotic frequency is a simple waveguide mode. However, by introducing the Peierls transition into this metal periodic structure, it can give rise to leaky modes or low-pass filtering effect. Since the frequency range of the waveguide adaptor used to feed the signals in experiments is X-Band, the arrangement of the whole one-dimensional metal diaphragm array needs to be designed. As a result, the guided and leaky wave phenomena can be observed in this frequency range, i.e., the one-dimensional metal periodic structure should be optimized. The total length of the waveguide selected here is 37.5 cm, and the number of triangular diaphragms is 75. The actual waveguide is made of metal aluminum.

**Figure 3:**
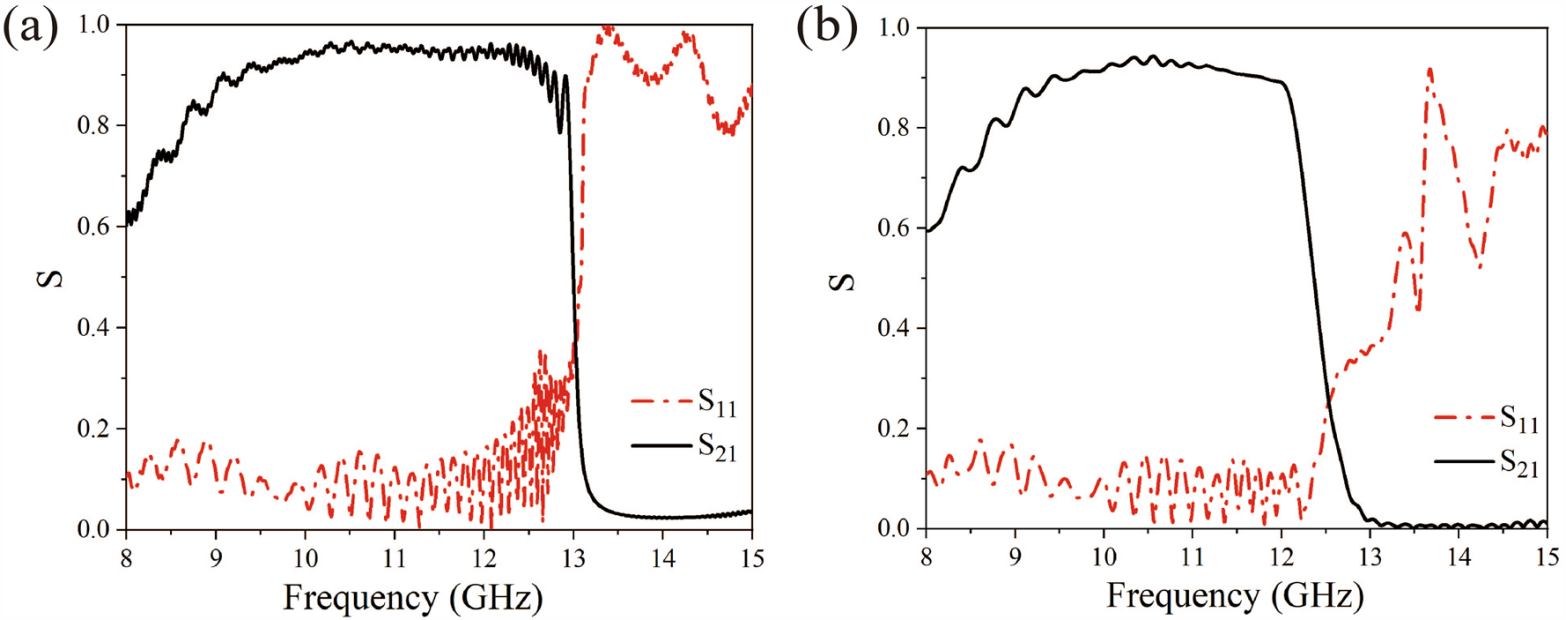
The simulation results of the S-parameters for the periodic metal structures: (**a**) the S-parameters of the 1-D triangular metal diaphragm array with lattice constant $d$ = 5.0 mm; (**b**) the S-parameters of the 1-D staggered triangular metal diaphragm array of lattice constant $d$ = 10.0 mm.

We shall discuss the transmission characteristics of the present waveguides. Figure 3a,b show the *S*-parameters numerical results of the waveguide structures that are presented in Fig. 1a,b, respectively, where the sizes of the waveguide port for feeding the signals is 22.8 mm wide and 10.2 mm long, and it is excited by using the TE_10_ mode. When the fundamental mode of the TM field for this waveguide adaptor is TM_11_, the frequency of the TM excitation is far beyond the frequency range under consideration.

**Figure 4:**
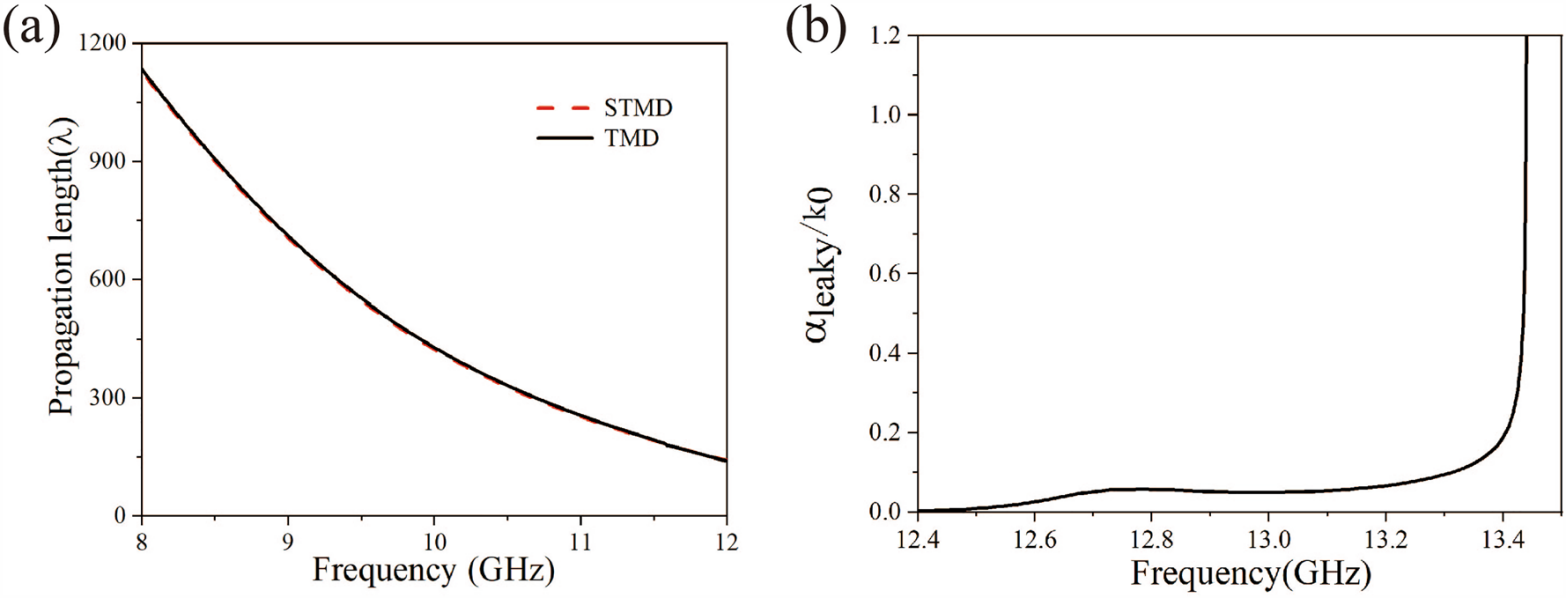
The propagation lengths and leaky loss of the periodic metal diaphragm structure: (**a**) the normalized propagation lengths of the 1-D triangular metal diaphragm array and the 1-D staggered triangular metal diaphragm array structure in the guided-wave frequency range; (**b**) the normalized imaginary part $\alpha_{leaky} /k_{0}$ of the 1-D staggered triangular metal diaphragm array in the leaky-wave frequency range.

Since the one-dimensional plasmonic waveguide is made of metal, the loss of the metal conductor and the electromagnetic wave energy radiation in the leaky wave frequency range must be taken into account. By using the scenario provided in Ref.^28^, the relation between the conductor ohmic loss and the frequency can be calculated within the framework of perturbation method, where the imaginary part of the propagation constant (due to conductor loss) is given by $\alpha_{loss}$ = $(P_{d} /P_{f} )/(2d)$. Here, $P_{d}$ is the metal loss per unit cell and $P_{f}$ is the total transmitted power. Propagation length is expressed as $L_{m}$ = $\alpha_{loss} /2$ = $(P_{f} /P_{d} )d$. The behavior of propagation length depending on frequency is shown in Fig. 4a. With the increase of frequency, the confinement of the electromagnetic field is increased, so that the propagation length decreases rapidly in high frequency region. In the frequency range of the leaky wave, the propagation constant is complex, i.e., $k_{z} = \beta + j\alpha_{leaky}$ with $\alpha_{leaky} = (P_{r} /P_{f} )/(2d)$, where $P_{r}$ is the transverse radiated power of the periodic metal structure. The frequency-dependent α is shown in Fig. 4b.

### Experimental measurement

**Figure 5:**
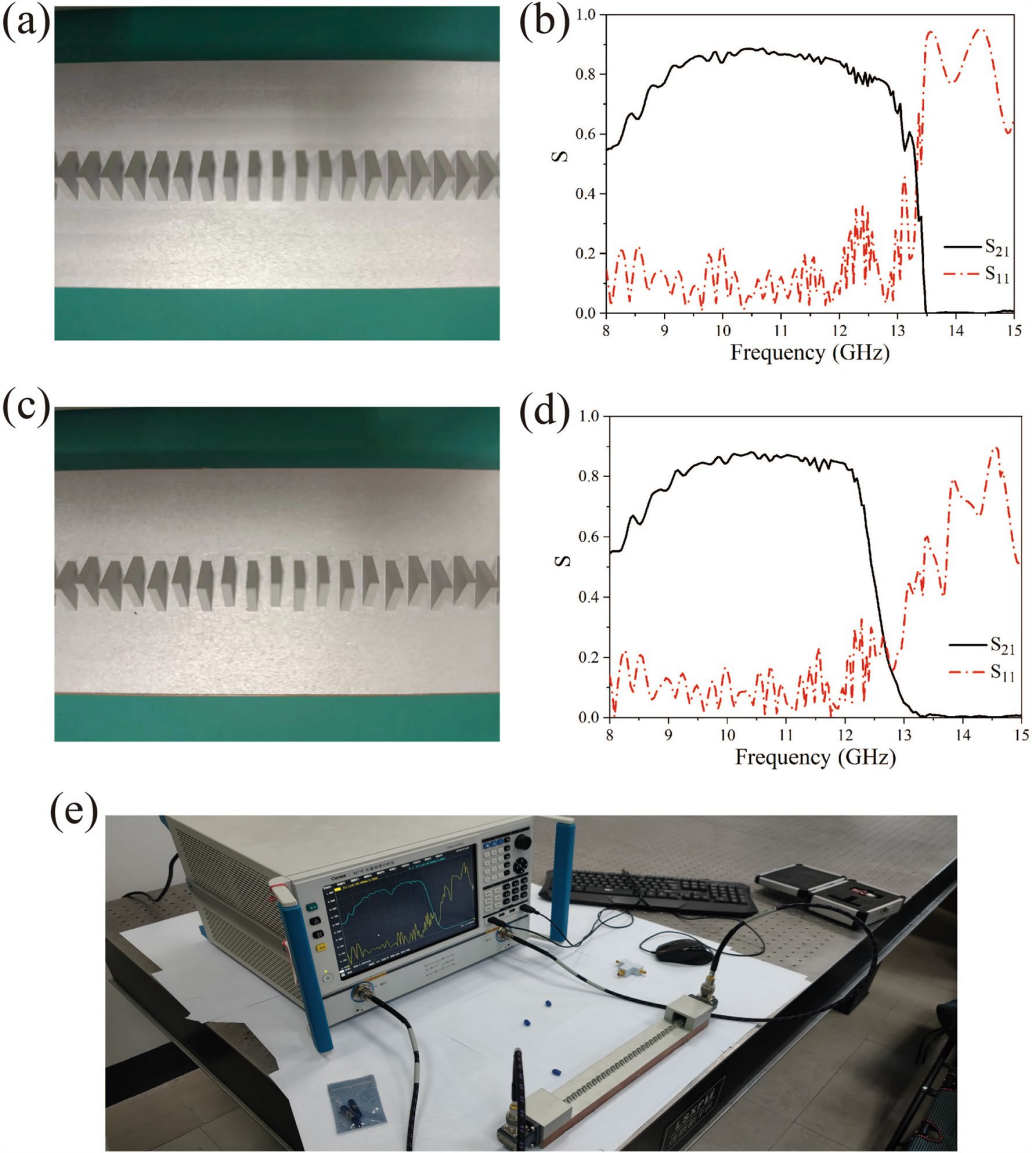
The experimental triangular periodic metal diaphragm structures and the measured *S*-parameters: (**a**) the triangular periodic metal diaphragm structure; (**b**) the measured *S*-parameters of a triangular periodic metal diaphragm structure; (**c**) the staggered triangular periodic metal diaphragm structure; (**d**) the measured S-parameters of a staggered triangular periodic metal diaphragm structure; (**e**) the photo of the waveguide *S*-parameters measurement system.

In order to verify the results of the above numerical simulation, the transmission characteristics of the triangular metal diaphragm periodic structures were measured in our experiments. According to the geometric structures shown in Fig. 1a,c, aluminum was used for fabricating the two plasmonic waveguides. The shape and size of the unit cells in the waveguide structure are the same as in the structure shown in Fig. 1, with a total length of 37.5 cm. A network analyzer was first used to measure the *S*-parameters of the triangular periodic metal diaphragm structures for determining the transmission characteristics of the whole waveguide. The actual structure of the triangular periodic metal diaphragm array sketched in Fig. 1a is shown in Fig. 5a. The measured *S*-parameters of the triangular metal diaphragm periodic array structure at frequencies ranging from 8 to 15 GHz is given in Fig. 5b. Here, the red dash-dotted line and the black solid line represent the curves of $S_{11}$ and $S_{21}$, respectively, which depend on the frequency. For example, the value of $S_{11}$ at the frequency 8.0 GHz is 0.14601 and oscillates rapidly in the frequency range 8.0–13.2 GHz. When the frequency of the signal begins to enter the edge of the bandgap, the value of $S_{11}$ of the signal suddenly increases rapidly with the frequency. For example,$S_{11}$ = 0.49732 at $f$ = 13.40 GHz and when the frequency increases to 13.56 GHz, the parameter $S_{11}$ = 0.94356, and this implies that the electromagnetic waves are almost totally reflected. It can be found that $S_{21}$ gradually increases from 0.54659 at $f$ = 8.00 GHz to 0.88732 at $f$ = 10.68 GHz, and approaches zero at $f$ = 13.56 GHz. A photo of the real staggered triangular periodic metal diaphragm structure after CNC machining is presented in Fig. 5c and the measured *S*-parameters of the staggered triangular metal diaphragm periodic structure is given in Fig. 5d. It can be observed that $S_{11}$ oscillates between 0.0 and 0.3 from frequency $f$ = 8.00 GHz to $f$ = 12.96 GHz and $S_{21}$ increases from 0.5445 at $f$ = 8.00 GHz to 0.8808 at $f$ = 10.12 GHz. The parameter $S_{21}$ starts with 0.70559 at $f$ = 12.28 GHz to 0.00555 at $f$ = 13.32 GHz, whereas $S_{11}$ does not immediately increase significantly with frequency, namely, in this frequency range, since the $S_{21}$ parameter decreases by two orders of magnitude and $S_{11}$ is less than 0.3, most of the electromagnetic energy has become a radiative mode. This, therefore, means that the electromagnetic waves are almost neither reflected nor transmitted. In other words, the electromagnetic waves have leaked into free space. The photo of the system we used to measure the *S*-parameters of the waveguide is shown in Fig. 5e. It can be found that the results of the experimental measurement of S-parameters are in good agreement with the theoretical calculation.

**Figure 6:**
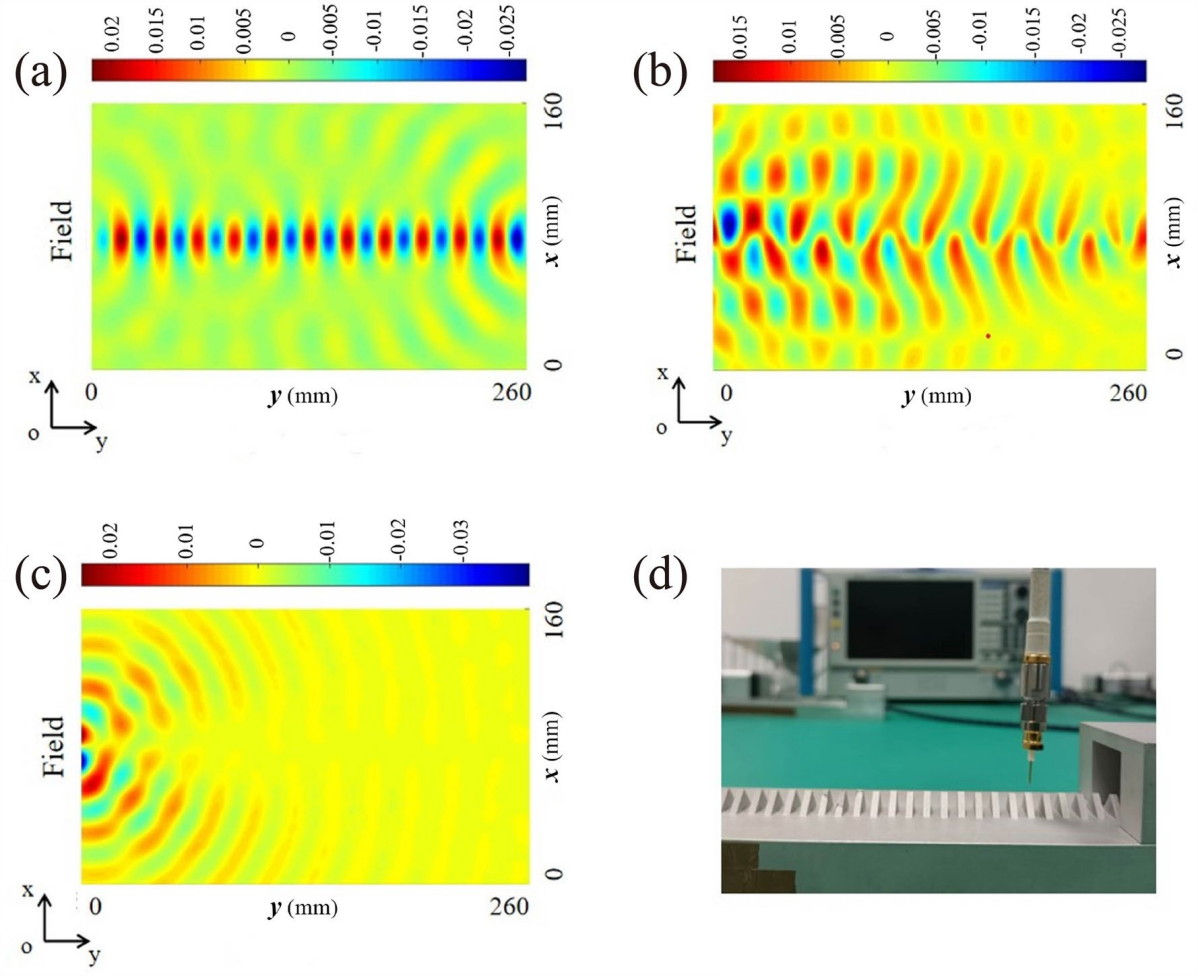
The near-field distribution of the staggered triangular periodic metal diaphragm structure, which was measured at (**a**) 11.00 GHz, (**b**) 12.49 GHz, (**c**) 13.50 GHz, and (**d**) is the near-field experiment photo.

On the other hand, by measuring the near-field distribution of the periodic metal structure, the field distribution of the periodic metal structure in the guided wave region and the leaky wave region can be directly observed. In Fig. 1c, there exists the three regions for the three modes (i.e., guided waves, leaky waves and band gap modes) in the metallic periodic structure for the considered frequency range, and the measurement results at frequencies $f$ = 11.00 GHz, 12.49 GHz and 13.50 GHz are shown in Fig. 6a–c, respectively. The photo of near-field detection setup is presented in Fig. 6d. Since the frequency $f$ = 11.00 GHz is located in the guided frequency range of Fig. 1c,a complete spatial distribution of the guided wave mode can be exhibited. As the frequency $f$ = 12.49 GHz is located in the frequency range of leaky wave mode in the periodic metal structure of Fig. 1c, the electromagnetic wave transmission in the metal periodic structure cannot maintain a complete electromagnetic mode spatial distribution, i.e., the electromagnetic field has leaked into the free space. Since the frequency $f$ = 13.50 GHz is located in the bandgap region of the periodic metal structure in Fig. 1c, the electromagnetic wave transmission in the metal periodic structure experiences total reflection effect.

**Figure 7:**
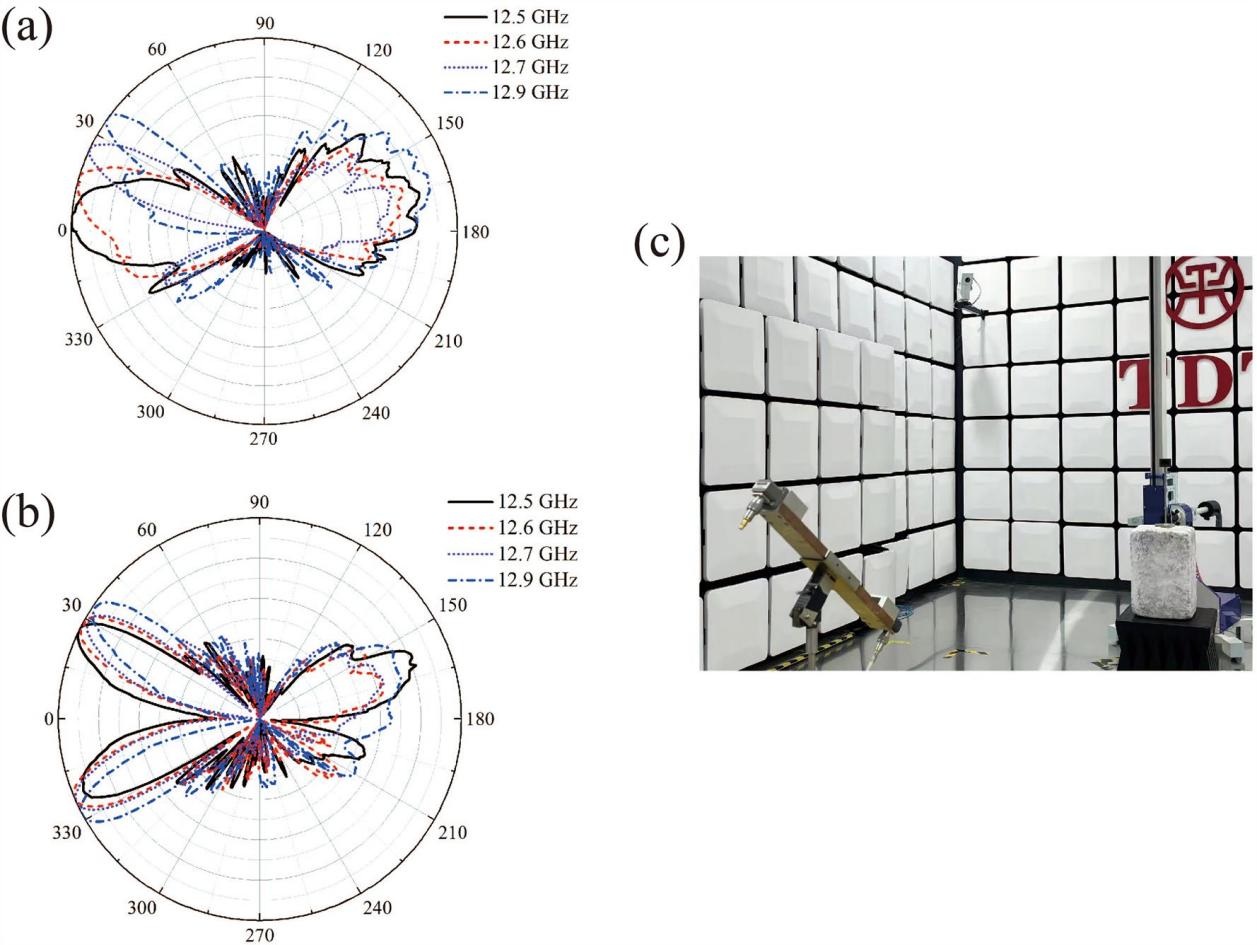
The far-field distribution of the staggered triangular periodic metal diaphragm structure in the leaky-wave frequency range measured in the experiment: (**a**) the main beam distribution depending on the elevation angle; (**b**) the field pattern and azimuth distribution of the main beam; (**c**) the measurement framework for the far-field radiation.

For the staggered triangular metal diaphragm periodic structure as shown in Fig. 1c, the leaky wave frequency range is located between $f$ = 12.2554 GHz and $f$ = 13.4370 GHz, and it is necessary to measure the far-field distribution of the staggered triangular metal diaphragm periodic structure of Fig. 1c by using a horn antenna. According to the simulation results, there are two main beams in the leaky wave frequency range, where the beam elevation angle increases gradually with the increasing frequency and the azimuth $\Delta \varphi$ of the two beams exhibits the same trend. Therefore, the appropriate leaky wave frequencies can be selected in the frequency band through the experimental measurement, namely, a total of four frequencies, i.e., 12.5 GHz, 12.6 GHz, 12.7 GHz, and 12.9 GHz, has been selected for the far-field radiation measurement. In Fig. 7a, we show the changes in the far-field distribution of the main beams with the elevation angles $\theta$ at the four frequencies, indicating that the main beam field distribution varies with frequency. It can be found that when the electromagnetic wave frequencies are chosen as $f$ = 12.5 GHz, 12.6 GHz and 12.9 GHz, the elevation angles of the main beam are $\theta =$ 4°, 15° and 36°, respectively. The two beams were swept as the frequency changes in the leaky-wave far field, and the angle $\Delta \varphi$ between the two beams increases with frequency. The measurement result has been shown in Fig. 7b. When the electromagnetic wave frequencies are chosen as $f$ = 12.5 GHz, 12.6 GHz and 12.9 GHz, the angles $\Delta \varphi$ between the two beams are 48°, 52^o^ and 61°, respectively. The present experimental measurements show that the angle between the two beams becomes large gradually with the increasing frequency. The measurements of the dispersion for the far fields are in line with the trends predicted by the numerical method. The measurement framework for the far-field radiation is shown in Fig. 7c.

Since most of the current microwave circuits have been replaced by planar circuits, the present type of periodic structures is still necessary as a system that exchanges energy between electrons and electromagnetic waves. The loss of metal conductors in THz band is not large, so it is still advantageous to scale down the one-dimensional metal diaphragm array to an open waveguide in THz band. In the solid state physics, the traditional Peierls transition, where the lattice constant of an atom lattice chain changes, leads to the modification in the electronic band structure. In the present electromagnetic analog of Peierls transition, however, what changes is the periodic artificial dielectric structure for propagating electromagnetic waves, and this gives rise to the photonic bandgap correction. Thus, the photonic Peierls transition involves the effect of modifying the bandgaps of one-dimensional metal periodic structures, which is essentially a role of manipulating the photonic bandgaps**.** In the literature, one-dimensional multi-layer film bandgap structures^30–32^, including the photonic and phononic crystals with various medium distribution such as tunable multichannel Fibonacci structure^32^, have been employed in some applied fields such as poliovirus sensing and greenhouse gas detection^30–33^. Some of the materials that are used to fabricate such multi-layer structures are superconducting nanomaterial-dielectric superlattices^34,35^. We expect that our scenario of tunable photonic bandgap structures could also find potential applications in sensor technology.

Since the propagation characteristic of one-dimensional metal periodic structures can be modified by Peierls transition, the frequency range of band gap and leakage wave can be changed by the number of metal films in the unit cell. Therefore, its applications may include the design of new low-pass filter and high directivity scanning antenna.

## Conclusion

In the present work of artificial electromagnetic structure design, we have introduced the concept of Peierls transition of solid physics into a 1-D periodic metal structure, where the photonic bandgaps can be adjusted on demand through the photonic analog of Peierls transition. If, taking a triangular metal diaphragm array as an example, a unit cell contains only one triangular metal diaphragm, the transmission bandwidth of a lowest-order mode can be determined by the cut-off and asymptotic frequencies. If, however, there is a slight relative displacement between adjacent triangular metal diaphragms (photonic Peierls transition), a unit cell will contain two triangular metal diaphragms, the lattice constant will be doubled, and the width of the first Brillouin zone will be halved. This will result in negative dispersion characteristics, providing a certain bandwidth of leaky wave modes. It can be seen that the easiest way for changing the electromagnetic wave transport properties of a periodic structure is to directly employ such a photonic Peierls transition in periodic structure itself. We expect that such a mechanism of photonic analog of Peierls transition would pave a new way for designing completely new waveguide device structures. The theoretical and experimental researches have been performed in this work, where the interesting photonic response involved in the electromagnetic analog of Peierls transition (e.g., the characteristics of leakage waves and filters can be generated through relatively small changes in one-dimensional metal periodic structures) is a major feature in the present paper.

## Methods

The dispersion curves of the 1-D triangular periodic metal diaphragm array structures were calculated by commercial FEM software (COMSOL). The far field distribution pattern of the periodic structures has been simulated by CST Microwave Studio. The performances of the fabricated 1-D triangular periodic metal diaphragm array structures such as transmission bandwidth, leaky radiation efficiency and far field radiation pattern have been tested through experiments.

## Data Availability

Data underlying the results presented in this paper are not publicly available at the present time but may be obtained from the corresponding authors upon reasonable request.
